# Electrocardiogram lead conversion from single-lead blindly-segmented signals

**DOI:** 10.1186/s12911-022-02063-6

**Published:** 2022-11-29

**Authors:** Sofia C. Beco, João Ribeiro Pinto, Jaime S. Cardoso

**Affiliations:** 1 Centre for Telecommunications and Multimedia, INESC TEC, Porto, Portugal; 2 Faculdade de Engenharia, Universidade do Porto, Porto, Portugal

**Keywords:** Autoencoder, Conversion, Deep learning, Electrocardiogram (ECG), Leads, U-Net

## Abstract

**Background:**

The standard configuration’s set of twelve electrocardiogram (ECG) leads is optimal for the medical diagnosis of diverse cardiac conditions. However, it requires ten electrodes on the patient’s limbs and chest, which is uncomfortable and cumbersome. Interlead conversion methods can reconstruct missing leads and enable more comfortable acquisitions, including in wearable devices, while still allowing for adequate diagnoses. Currently, methodologies for interlead ECG conversion either require multiple reference (input) leads and/or require input signals to be temporally aligned considering the ECG landmarks.

**Methods:**

Unlike the methods in the literature, this paper studies the possibility of converting ECG signals into all twelve standard configuration leads using signal segments from only one reference lead, without temporal alignment (blindly-segmented). The proposed methodology is based on a deep learning encoder-decoder U-Net architecture, which is compared with adaptations based on convolutional autoencoders and label refinement networks. Moreover, the method is explored for conversion with one single shared encoder or multiple individual encoders for each lead.

**Results:**

Despite the more challenging settings, the proposed methodology was able to attain state-of-the-art level performance in multiple target leads, and both lead I and lead II seem especially suitable to convert certain sets of leads. In cross-database tests, the methodology offered promising results despite acquisition setup differences. Furthermore, results show that the presence of medical conditions does not have a considerable effect on the method’s performance.

**Conclusions:**

This study shows the feasibility of converting ECG signals using single-lead blindly-segmented inputs. Although the results are promising, further efforts should be devoted towards the improvement of the methodologies, especially the robustness to diverse acquisition setups, in order to be applicable to cardiac health monitoring in wearable devices and less obtrusive clinical scenarios.

## Introduction

**Fig. 1:**
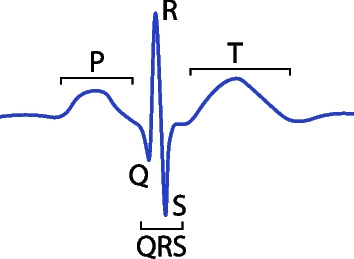
Illustration of a sample ECG heartbeat with its characteristic waveforms

The electrocardiogram (ECG) is the measurement of electrical potentials that make the heart contract and relax as intended. It is composed of a cyclic repetition of five characteristic and easily recognisable waveforms P, Q, R, S, and T (see Fig. 1). The morphologies of the ECG signal and these waveforms depend on the location of the electrodes used for acquisition: different electrode placement results in different perspectives over the heart [1]. For medical purposes, the standard configuration acquires the ECG over twelve leads for more information, but it requires ten electrodes placed on the patient’s arms, legs, and chest. Using fewer electrodes allows for more comfortable and inexpensive acquisitions, at the expense of certain leads that could be ideal for a more accurate diagnosis of certain conditions.

To get the best of both worlds, researchers have proposed methods for the automatic interlead conversion of ECG signals [2–6]. These transform short ECG segments to mimic other perspectives, using acquired leads to reconstruct any leads that were not recorded. However, these methods still present limited applicability, since they typically require multiple leads as input. Even the most advanced methods [4, 5], that only use one input lead, still require the inputs to be single heartbeat segments aligned in time, which makes them dependent on separate processes and, overall, less flexible and robust. Moreover, despite the advances in ECG synthesis from reduced lead sets, converted signals may still suffer from slight amplitude changes, small distortions, or loss of finer details that could be essential for certain tasks [7]. Additional file 1: Fig S1

**Fig. 2:**
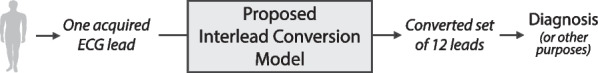
Simple schema of the proposed method, which receives single-lead ECG signals and delivers reconstructed 12-lead signals to be used for diagnosis or other purposes

This paper presents a study on the feasibility of ECG interlead conversion using short segments from just one limb lead without any kind of temporal alignment (blindly-segmented). With such input, the proposed methodology is trained to reconstruct other leads as faithfully as possible. This aims to open up new possibilities for more comfortable ECG acquisition in clinical scenarios or wearable devices without giving up the benefits of multi-lead recordings for medical diagnosis (see Fig. 2).

The proposed methodology, based on deep learning encoder-decoder structures, is explored for interlead conversion using either lead II or lead I (limb leads) signals as reference, and using a single shared encoder or an individual encoder for each target lead. Beyond the training and testing on the widely used PTB database, the conversion models are evaluated on cross-database scenarios with the INCART and PTB-XL databases. Additionally, the clinical annotations of the PTB-XL database are also used for a differential performance evaluation in the presence of medical conditions, and an evaluation of the performance of a state-of-the-art diagnosis model with original *vs*. reconstructed signals. The code used for this work is available online^1^.

## Related work

At the onset of research on interlead conversion, methodologies commonly required several leads as reference for robust lead reconstruction. Zhu et al. [8] performed a preliminary study on the conversion of ambulatory ECG recordings into standard 12-lead ECG signals using lead-field theory and the least-squares method. Nelwan et al. [9] learned generic and patient-specific linear regression coefficient templates to reconstruct up to four missing leads with high correlation results.

Later, Yoshida et al. [10] used 12 lead acquisitions to synthesise additional leads (right ventricular leads V3R, V4R, and V5R and posterior chest leads V7, V8, and V9) which provide important information for the diagnosis of acute myocardial infarction. Their algorithm was based on the transfer coefficient estimated from the learning data. Additional file 2: Fig S2

Silva et al. [2] developed three methods for obtaining the Frank leads using the 12 standard leads as reference: the Kors Quasi-Orthogonal method, the Kors Linear Regression method, and the Dower Inverse Matrix. The conversion was successful for signals from healthy subjects but presented limitations on signals from subjects with pathologies. The recent work by Smith *et al.* [6] was one of the first to use machine learning techniques for interlead conversion. They used a focused time-delay neural network (FTDNN), which is well suited for time series prediction. However, their methodology required seven input leads (all limb leads and V1).Additional file 3: Fig S3

Atoui et al. [11] used ensembles of fully-connected neural networks to learn to synthesise V1, V3, V4, V5, and V6 heartbeats from three-lead inputs (I, II, and V2). Schreck et al. [12] performed the first study on the synthesis of the entire set of 12 standard leads and scalar 3-lead derived vectorcardiogram from just three measured leads. Their proposed methodology used nonlinear optimisation to construct a universal patient transformation matrix. Hansen et al. [13] applied linear generic and subject-specific transforms to convert recordings from adhesive patch-type ECG monitors to the standard 12-lead ECG signals. In [14, 15], researchers also explored personalised statistically determined linear transforms and went on to achieve improved results. Additional file 4: Fig S4

Lee et al. [16] proposed methods based on linear regression and artificial neural networks to reconstruct the 12 standard leads from subsets of 35 channels acquired using one single large patch covering the subject’s chest. Although accurate, the method is arguably incompatible with scenarios focused on ease of use and patient/user comfort. Similarly, Grande-Fidalgo *et al.* [17] used linear regression and fully-connected networks to reconstruct the entire set of twelve standard leads from a subset of just three input leads. Sohn et al. [3] used long short-term memory (LSTM) networks to accomplish the reconstruction of the twelve ECG standard leads from a three-lead patch-type device. Their results show their method was able to correctly retain pathological abnormalities from medical conditions on the reconstructed signals.

The work of Lee et al. [4] was one of the few that studied the synthesis of standard leads using only one reference lead. In their study, chest leads (V1 to V6) were synthesised from lead II using a generative adversarial network (GAN). However, input segments had to be single heartbeats, aligned according to the R-peaks, which decreases the difficulty but also the applicability of the method. Matyschik et al. [5] developed patient-specific models to more accurately reconstruct eleven missing ECG signals from a single available lead of the standard 12-lead system. However, the reference lead was either V1, V2, or V3 which, being chest leads, do not enable the usage in less obtrusive setups which would preferentially use limb leads.

In this work, we explore the more challenging scenario of reconstructing the entire set of twelve standard leads using only one reference lead. Moreover, the reference signals are blindly-segmented (without any kind of temporal alignment) and pertain to one of the limb leads to allow for applications on the least obtrusive setups. Our main goal is to assess whether it is possible to reconstruct the electrocardiogram signal in such challenging scenarios and discuss the next steps towards the use of interlead conversion in less obtrusive clinical setups and wearable devices.Additional file 5: Fig S5

## Methodology

### General overview

The proposed methodology for interlead ECG conversion follows the encoder-decoder structure typically used for deep image segmentation. The encoder receives an input signal and processes it to create a compressed representation that retains relevant information for the task at hand. The decoder receives this representation and processes it so that the output matches the ground-truth as closely as possible. Here, the input to the encoder is a short ECG segment of one lead (X) and the ground-truth is the corresponding segment in a different lead (Y). Thus, the encoder is in charge of selecting the information from X that is needed for Y, and the decoder will use that information to reconstruct the corresponding lead Y signal.Additional file 6: Fig S6

### Model architectures

The general encoder-decoder structure allows for diverse specific model architectures. This work focuses on the U-Net model, a fully convolutional architecture that has found many applications related to semantic segmentation and can also be adapted for the task of ECG lead conversion. Additional file 7: Fig S7

#### U-Net

**Fig. 3:**
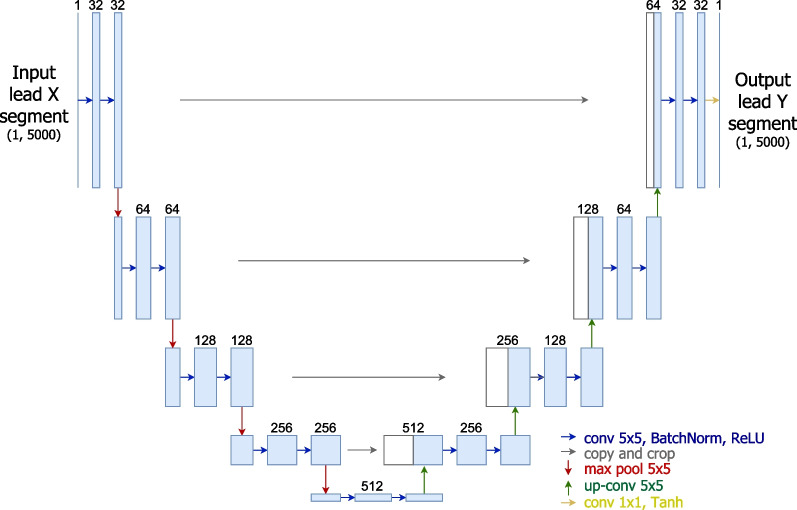
Overview schema of the main U-Net architecture used in this work for lead conversion

The U-Net was initially proposed by Ronneberger et al. [18] as a tool for biomedical image segmentation. In this work, the implemented architecture (see Fig. 3) receives an input segment of lead X, which initially goes through a chain of three sequential blocks, each with half the signal resolution of the previous block. Each block includes two convolutional layers (each followed by batch normalisation and ReLU activation) and ends with a max-pooling layer.

Between the encoder and the decoder, two convolutional layers compose the latent space or bottleneck block, which corresponds to the maximum point of information compression. The decoder mirrors the encoder in its structure, with three similar blocks composed of an upsampling layer and two transposed convolutional layers. The last transposed convolutional layer outputs a single-channel signal whose size corresponds to the input segment. The activation function of this last layer is the hyperbolic tangent for an output signal with amplitudes in $[-1, 1]$.

One aspect of the U-Net which is often cited as the key to its widespread success is the skip-connection. U-Nets typically include skip-connections between corresponding blocks on the encoder and the decoder. This means the feature maps from the encoder blocks are directly routed to the corresponding decoder blocks, allowing the model to propagate context information from multiple resolutions between the encoder and the decoder for higher flexibility. Additional file 8: Fig S8

#### Convolutional autoencoder (AE)

**Fig. 4:**
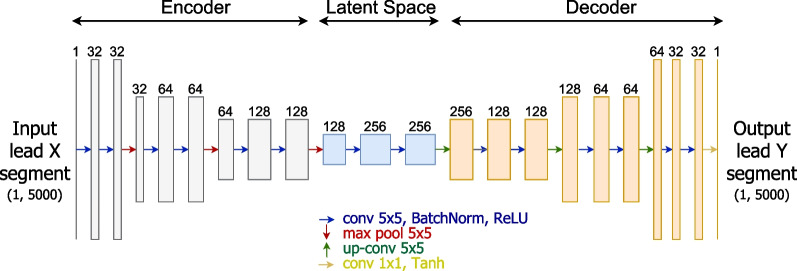
Overview schema of the convolutional autoencoder (AE) architecture

Beyond the aforementioned U-Net architecture, adapted for unidimensional signal inputs, we also explore a convolutional autoencoder (AE, see Fig. 4). Its architecture is very similar to the U-Net, albeit without skip-connections. As a result, the structure is simplified, when compared to the U-Net, and the latent representation sent from the encoder to the decoder is smaller. Experiments with the AE architecture aim to assess if the skip-connections are essential for the task at hand or if the simplified structure could avoid overfitting and bring performance benefits.

#### Label refinement network (LRN)

**Fig. 5:**
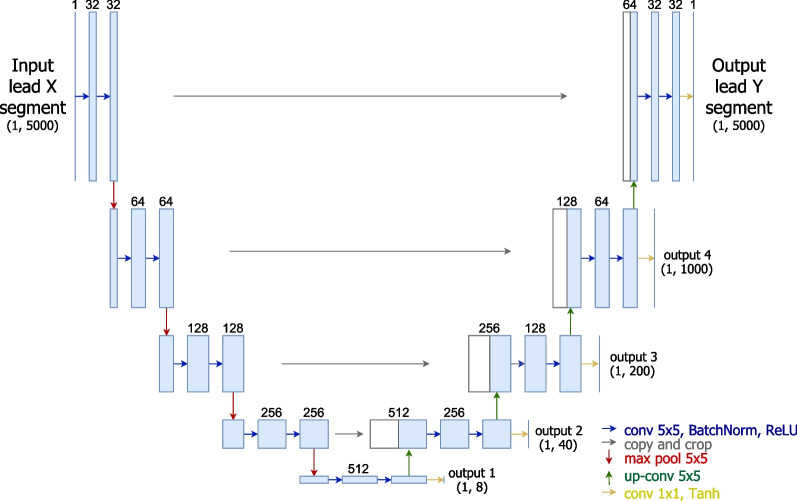
Overview schema of the architecture based on label refinement networks (LRN)

The third architecture explored in this work was based on Label Refinement Network (LRN, see Fig. 5) was originally proposed by Islam et al. [19] for semantic image segmentation. Its architecture is identical to the aforementioned U-Net. The singularity of the LRN lies in the supervision strategy: while the U-Net only uses the output of the last decoder block in the reconstruction loss, the LRN computes the loss at the outputs of every decoder block. This results in supervision at several resolution levels, leading the decoder to offer a coarse reconstruction right after the first block, which should be gradually refined by the subsequent blocks for improved results at higher resolutions. Experiments with the LRN architecture aim to assess if the multi-level resolution could bring improved performance to the task of signal lead conversion as they have for semantic segmentation.

### Shared *vs.* individual encoders

The conversion of one lead into multiple missing leads requires multiple decoders - each one will fulfil the task of reconstructing its respective lead based on the compressed latent representation. In the case of the encoder, however, it is possible to have a single one whose output will be shared by all decoders or have multiple encoders, each one dedicated to one individual decoder.

In this work, we explore both possibilities for 12-lead reconstruction - using one shared encoder connected to all 11 decoders, for all 11 output leads except the one corresponding to the input, or using one individual encoder for each of the 11 decoders. Using individual encoders grants more flexibility to each lead conversion process, as each encoder will be able to learn a unique way to obtain compressed representations and each encoder-decoder pair will work independently from all others. On the other hand, using one shared encoder results in a much lighter and faster algorithm and the added simplicity may contribute towards avoiding overfitting.

## Experimental setup

### Data

The experiments conducted in this work used mainly the data provided in the PTB Diagnostic ECG Database [20], available on Physionet [21]. The PTB database includes data from 16 channels, including all 12 standard leads, sampled at 1 kHz. It contains a total of 549 records from 290 individuals, with one to five records per subject. Recordings were cropped into segments of 5 s (5000 samples). A second-order Butterworth bandpass filter with cut-off frequencies $f_c = [1, 40]\ Hz$ was applied to each segment to remove noise while retaining the most useful ECG information. The amplitudes of the *n* values of each signal *x* were then min-max normalised to the interval $[-1, 1]$ following the equation:

1 $$\begin{aligned} x_n = 2\times \frac{x_n-x_{min}}{x_{max} - x_{min}} - 1. \end{aligned}$$

The data from PTB was divided into train and test sets, with approximately $63\%$, $7\%$ and $30\%$ of the segments, respectively, for a total of 7086, 787, and 3509 ECG segments for each set. For a more thorough and challenging evaluation, subjects are divided between the train/validation and test sets: the latter had recordings from subjects 1 to 50 while the former had recordings from subjects 51 to 290.

The INCART database (officially the St. Petersburg INCART 12-lead Arrhythmia Database), also available on Physionet, was used to test the performance of trained models on cross-database scenarios. This database contains 75 Holter recordings from 32 subjects undergoing tests for coronary artery diseases. Each record is 30 minutes long and contains twelve standard leads sampled at 257 Hz. Recordings from this database were resampled to 1 kHz and processed as described above for PTB.

The PTB-XL database [22, 23], created by the same team as the PTB, includes 21837 clinical ECG recordings from a total of 18885 patients. Each recording is 10 seconds long, includes all twelve standard ECG leads, and is originally sampled at 500 Hz. The waveforms were annotated by up to two cardiologists, who assigned annotations to each record. The 71 possible annotation statements have been clustered into five superclasses: NORM (normal ECG), MI (myocardial infarction), STTC (ST/T change), CD (conduction disturbance), and HYP (hypertrophy). This dataset was originally created for the training and evaluation of automatic ECG interpretation algorithms but also shows great promise for the development of lead conversion algorithms. In this work, we take advantage of expert clinical annotations to study the effect of medical conditions on the quality of the lead conversion results. From the total of 21837 recordings, we selected the 16272 that did not have conflicting superclass annotations. From each recording, the first 5 seconds were cropped, resampled to 1 kHz, and processed as described above for PTB.

### Model training and evaluation

The models were trained using the *l*1-loss between the model outputs and the corresponding ground-truth signals as the objective function. The *l*1 was chosen empirically as it allowed the model to learn most adequately both the overall morphology of the signals and their finer details. The Adam optimiser was used with an initial learning rate of $1\times 10^{-3}$, over a maximum of 500 epochs with batch size 32 (shared encoder) or 16 (individual encoder) and early stopping patience of 50 epochs.

To compare lead conversions with the corresponding measured ground-truth signals, this work used the following metrics: the average and median Pearson correlation coefficient (*r*, used in the majority of the related literature), the average root mean square error (RMSE), and the average Structural Similarity Index Measure (SSIM).

## Results

### Architecture comparison

To compare the selected architectures, the first experiment entailed the one-to-one lead conversion from II to I, two of the most used ECG leads for medical purposes (see Table 1). According to the results, the U-Net performs better than both alternatives AE and LRN. Although the AE achieves the same median *r* as the U-Net, the average *r* is lower, meaning that the least successful results are generally worse with the AE than the U-Net. Following the results of this comparison, subsequent experiments focus solely on the U-Net architecture.

**Table 1:** Comparison of encoder-decoder architectures on one-to-one lead conversion

Model	*r* (avg.)	*r* (med.)
U-Net	0.69	0.78
Autoencoder	0.67	0.78
LRN	0.65	0.75

### One-to-all leads conversion

**Table 2:** Average correlation between lead II signals and the remaining leads on the PTB, INCART, and PTB-XL databases

	I	III	aVR	aVL	aVF	V1	V2	V3	V4	V5	V6
PTB	0.45	0.36	−0.71	0.01	0.77	−0.34	−0.20	0.00	0.28	0.72	0.81
INCART	0.46	0.80	−0.86	−0.49	0.95	−0.45	−0.19	0.25	0.65	0.82	0.77
PTB-XL	0.70	0.31	0.25	−0.82	0.83	−0.44	−0.04	0.37	0.68	0.81	0.84

Not all leads can be converted equally: the correlation between leads depends on their perspectives of the heart. Table 2 presents an overview of the average correlation between lead II and the remaining eleven standard leads, computed using the PTB, INCART, and PTB-XL test segments. Specifically for the PTB data, one can observe that some leads such as aVF or aVR are highly (positively or negatively) correlated with lead II. On the other hand, aVL is almost orthogonal. Hence, one should expect aVL to be much harder to accurately convert from lead II than aVF or aVR, since the former shares much less information with lead II than the latter.

**Table 3:** Test results of the U-Net used for multi-lead conversion from lead II, with shared or individual encoders

	Shared encoder				Individual Encoders			
Lead	*r* (avg.)	*r* (med.)	RMSE	SSIM	*r* (avg.)	*r* (med.)	RMSE	SSIM
I	0.67	0.73	0.28	0.28	0.66	0.71	0.29	0.26
III	0.56	0.65	0.29	0.63	0.56	0.70	0.29	0.64
aVR	0.89	0.95	0.12	0.92	0.90	0.95	0.12	0.92
aVL	0.47	0.58	0.36	0.15	0.47	0.61	0.36	0.16
aVF	0.81	0.88	0.20	0.64	0.83	0.90	0.19	0.64
V1	0.77	0.84	0.20	0.87	0.80	0.86	0.18	0.88
V2	0.66	0.72	0.26	0.80	0.67	0.75	0.24	0.81
V3	0.56	0.62	0.33	0.65	0.59	0.66	0.31	0.66
V4	0.50	0.57	0.36	0.43	0.50	0.58	0.36	0.44
V5	0.70	0.77	0.27	0.36	0.74	0.80	0.26	0.40
V6	0.79	0.87	0.21	0.49	0.80	0.87	0.21	0.49

**Fig. 6:**
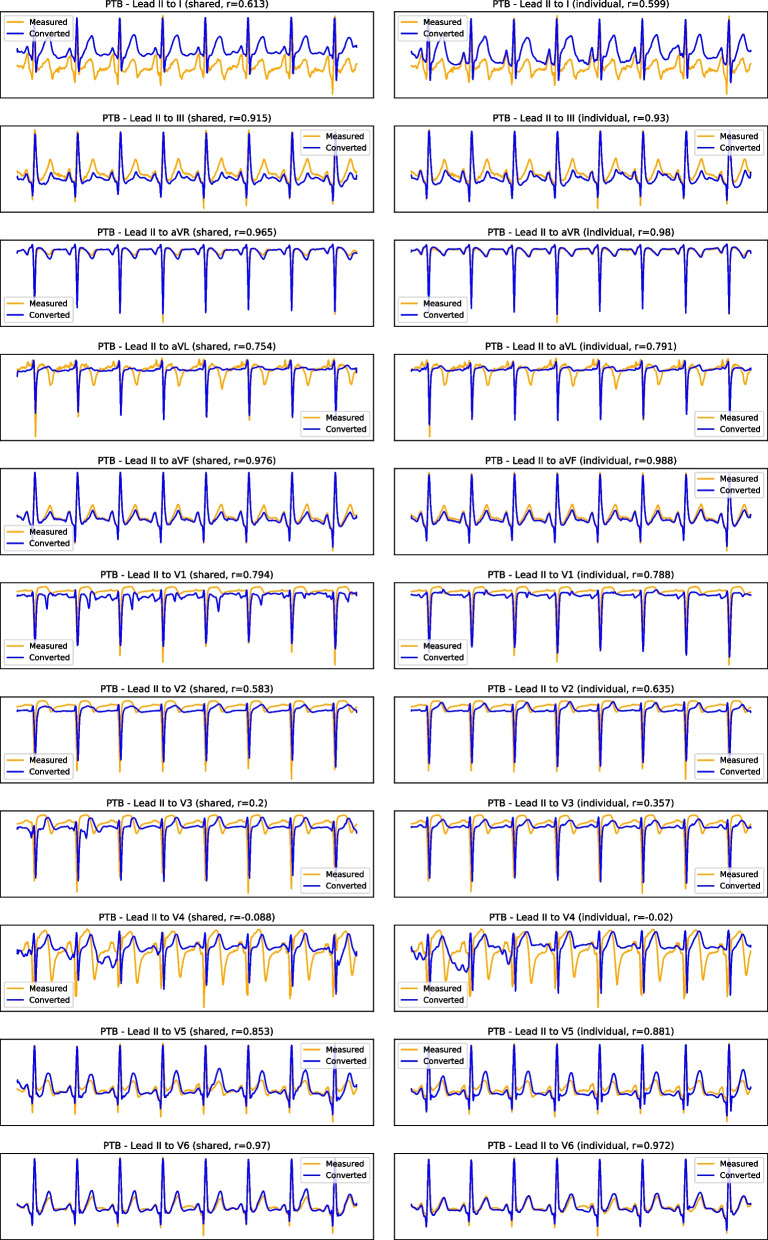
Example result of lead II to all conversion on the PTB test dataset (each row depicts one converted lead, with the shared encoder on the left column and individual encoders in the right column; the horizontal axis represents time, while the vertical axis corresponds to the normalised signal amplitude)

This is verified in the results for multi-lead conversion on the PTB database (see Table 3). Conversion from lead II to aVF, aVR, and V6 consistently offer good results, while the conversions to aVL, lead I, or V4 were overall the least successful. This behaviour is also visible in the example of Fig. 6,^2^ where the model is unable to capture the finer details of the signals in lead aVL and leads V1-V4. The opposite happens in lead III, aVF, V6, and especially aVR, where the model was consistently able to capture the morphological details of the signals.

**Table 4:** Average correlation between lead I signals and the remaining leads on the PTB, INCART, and PTB-XL databases

	II	III	aVR	aVL	aVF	V1	V2	V3	V4	V5	V6
PTB	0.45	−0.49	−0.82	0.82	−0.05	−0.47	−0.21	0.03	0.30	0.64	0.68
INCART	0.46	0.02	−0.62	0.32	0.26	−0.36	0.01	0.11	0.35	0.51	0.44
PTB-XL	0.70	−0.24	0.77	−0.86	0.32	−0.54	−0.06	0.33	0.63	0.80	0.83

**Fig. 7:**
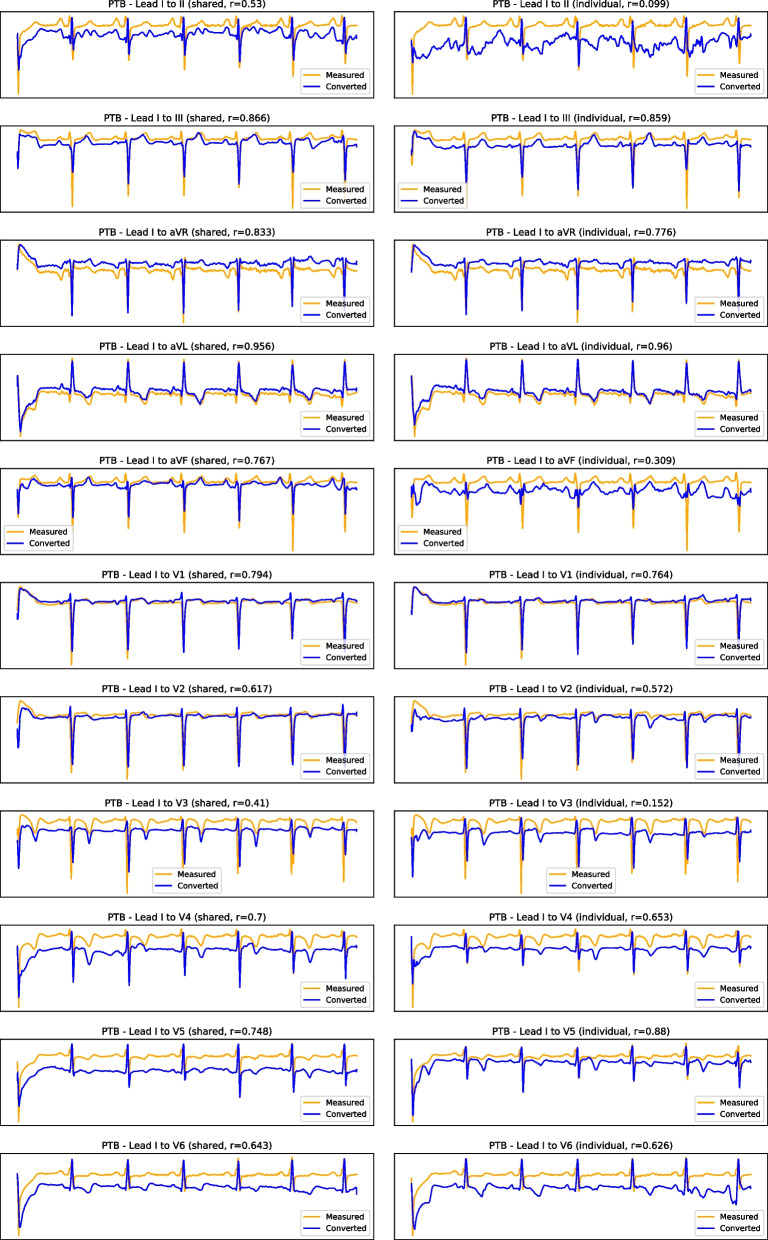
Example result of lead I to all conversion on the PTB test dataset (each row depicts one converted lead, with the shared encoder on the left column and individual encoders in the right column; the horizontal axis represents time, while the vertical axis corresponds to the normalised signal amplitude)

As for lead I, Table 4 presents the average correlation between this lead and the remaining eleven standard leads on the PTB, INCART, and PTB-XL test segments. As with lead II, lead I is more correlated (positively or negatively) with certain leads, such as aVR, aVL, or V6, while it is almost orthogonal with aVF or V3. As such, one can observe, in Table 5, that the proposed methodology obtains better performance with aVR and aVL while struggling to convert from lead I to lead aVF. The same can be observed in Fig. 7: for aVR and aVL, the model is able to correctly capture the target morphology, while the reconstructions of aVF and V3-V6 are largely unsuccessful.

**Table 5:** Test results of the U-Net used for multi-lead conversion from lead I, with shared or individual encoders

Lead	Shared encoder				Individual encoders			
	*r* (avg.)	*r* (med.)	RMSE	SSIM	*r* (avg.)	*r* (med.)	RMSE	SSIM
II	0.49	0.54	0.37	0.17	0.50	0.55	0.36	0.19
III	0.44	0.49	0.35	0.53	0.46	0.52	0.35	0.55
aVR	0.89	0.92	0.14	0.92	0.90	0.93	0.13	0.93
aVL	0.76	0.84	0.25	0.45	0.77	0.85	0.26	0.46
aVF	0.26	0.29	0.43	0.28	0.28	0.32	0.42	0.27
V1	0.81	0.88	0.18	0.88	0.79	0.88	0.19	0.88
V2	0.73	0.80	0.23	0.81	0.70	0.77	0.25	0.80
V3	0.67	0.73	0.28	0.70	0.67	0.74	0.29	0.68
V4	0.59	0.65	0.33	0.48	0.62	0.71	0.32	0.48
V5	0.62	0.73	0.30	0.31	0.64	0.73	0.30	0.28
V6	0.66	0.75	0.26	0.39	0.67	0.77	0.26	0.39

From the example result in Fig. 7, one can also identify a shortcoming of the proposed methodology: the occasional offsets between the baseline of the measured and converted signals. We suspect this is due to the min-max normalisation of the signals, drawing them into the $[-1, 1]$ amplitude range. Alternatives to this normalisation, such as standard normalisation, should be further investigated.

Using either lead as a reference, there is apparently no considerable or consistent difference between using one single shared encoder or using an individual encoder for each target lead. As for processing time, the model with individual encoders took an average of 15.28 ms per signal in training mode and 5.62 ms in inference mode. The model with a shared encoder was faster, as expected, requiring an average of 9.70 ms per signal on training mode and 4.16 ms on inference mode^3^.

### Comparison with the state-of-the-art

For a comparison with the state-of-the-art, we implemented the method recently proposed by Grande-Fidalgo et al. [17] as a baseline. This method is based on a simple fully-connected model that receives each signal point’s amplitude in three reference leads as inputs and returns the same point’s amplitude in all twelve leads. Here, we adapt the methodology so it receives signal point amplitudes from one single lead (leads I or II), to exactly match the evaluation conditions of the proposed method.

Unlike what has been reported in [17], the baseline was not successful in learning to retrieve the entire set of leads from just one reference lead. In fact, across all leads, the average test *r* of this method ranged from $-0.005$ to 0.002, considerably worse than the proposed methodology.

### Cross-database evaluation

The cross-database tests aimed to assess the behaviour of the proposed methodology on more diverse scenarios. Here, the models used were the same as in the previous experiments (trained with PTB data), and the evaluation was conducted using data from the INCART and PTB-XL databases.

For both INCART and PTB-XL, some differences in interlead correlations can be observed when compared to PTB (see Table 2 and Table 4). This can be explained due to the different acquisition setups, especially the positioning of the electrodes, which potentially causes each lead to offer a different perspective.

**Table 6:** Cross-database test results for INCART conversion from lead II

Lead	Shared encoder				Individual encoders			
	*r* (avg.)	*r* (med.)	RMSE	SSIM	*r* (avg.)	*r* (med.)	RMSE	SSIM
I	0.46	0.51	0.34	0.18	0.44	0.50	0.35	0.16
III	0.57	0.63	0.28	0.49	0.57	0.63	0.29	0.45
aVR	0.91	0.95	0.11	0.92	0.92	0.95	0.11	0.92
aVL	0.13	0.11	0.41	0.33	0.10	0.09	0.44	0.27
aVF	0.88	0.93	0.15	0.68	0.92	0.95	0.13	0.71
V1	0.63	0.79	0.23	0.82	0.65	0.82	0.23	0.83
V2	0.53	0.64	0.26	0.73	0.55	0.69	0.27	0.74
V3	0.42	0.51	0.33	0.56	0.42	0.53	0.34	0.55
V4	0.52	0.59	0.35	0.28	0.52	0.60	0.35	0.30
V5	0.73	0.80	0.25	0.31	0.74	0.80	0.25	0.29
V6	0.73	0.83	0.23	0.42	0.72	0.81	0.24	0.39

**Table 7:** Cross-database test results for INCART conversion from lead I

Lead	Shared encoder				Individual encoders			
	*r* (avg.)	*r* (med.)	RMSE	SSIM	*r* (avg.)	*r* (med.)	RMSE	SSIM
II	0.35	0.37	0.37	0.18	0.36	0.38	0.37	0.19
III	0.17	0.19	0.41	0.27	0.17	0.19	0.43	0.28
aVR	0.65	0.74	0.23	0.81	0.67	0.78	0.22	0.83
aVL	0.40	0.49	0.32	0.52	0.36	0.46	0.35	0.47
aVF	0.17	0.15	0.41	0.23	0.17	0.14	0.41	0.22
V1	0.55	0.62	0.25	0.78	0.57	0.64	0.24	0.79
V2	0.50	0.57	0.27	0.73	0.50	0.56	0.28	0.73
V3	0.35	0.35	0.37	0.46	0.36	0.37	0.37	0.44
V4	0.27	0.26	0.43	0.16	0.34	0.36	0.41	0.19
V5	0.46	0.51	0.36	0.11	0.45	0.53	0.35	0.11
V6	0.44	0.49	0.34	0.22	0.45	0.52	0.34	0.21

For INCART (see Table 6 and Table 7), the overall quality of the results is inferior to that with PTB. Despite these metrics, it is noticeable in the reconstruction plots (available in the Additional file material) that both reference leads can offer good conversion results in some leads, especially with lead II. Using this lead as reference, the proposed methodology is relatively good at converting most leads except I, V2, and V3.

**Table 8:** Cross-database test results for PTB-XL conversion from lead II

Lead	Shared encoder				Individual encoders			
	*r* (avg.)	*r* (med.)	RMSE	SSIM	*r* (avg.)	*r* (med.)	RMSE	SSIM
I	0.74	0.80	0.25	0.31	0.72	0.79	0.26	0.29
III	0.44	0.50	0.32	0.50	0.45	0.52	0.32	0.50
aVR	−0.38	−0.53	0.72	0.09	−0.39	−0.55	0.72	0.09
aVL	−0.33	−0.44	0.64	0.23	−0.33	−0.45	0.67	0.17
aVF	0.83	0.90	0.19	0.61	0.84	0.92	0.19	0.62
V1	0.79	0.87	0.17	0.91	0.81	0.89	0.16	0.91
V2	0.71	0.79	0.22	0.84	0.72	0.82	0.21	0.85
V3	0.61	0.68	0.30	0.66	0.62	0.70	0.30	0.66
V4	0.64	0.71	0.31	0.31	0.66	0.74	0.32	0.32
V5	0.79	0.86	0.22	0.37	0.80	0.87	0.23	0.39
V6	0.85	0.91	0.18	0.58	0.85	0.91	0.18	0.58

**Table 9:** Cross-database test results for PTB-XL conversion from lead I

Lead	Shared encoder				Individual encoders			
	*r* (avg.)	*r* (med.)	RMSE	SSIM	*r* (avg.)	*r* (med.)	RMSE	SSIM
II	0.60	0.66	0.33	0.23	0.62	0.70	0.31	0.26
III	0.31	0.34	0.38	0.43	0.33	0.38	0.37	0.45
aVR	−0.61	−0.76	0.74	0.09	−0.62	−0.78	0.74	0.09
aVL	−0.63	−0.75	0.75	0.11	−0.66	−0.79	0.76	0.08
aVF	0.29	0.31	0.41	0.23	0.32	0.36	0.40	0.24
V1	0.79	0.86	0.16	0.91	0.81	0.88	0.15	0.91
V2	0.71	0.78	0.22	0.84	0.70	0.77	0.23	0.83
V3	0.65	0.72	0.29	0.64	0.67	0.76	0.28	0.66
V4	0.62	0.72	0.32	0.30	0.69	0.80	0.30	0.32
V5	0.76	0.85	0.24	0.35	0.76	0.86	0.25	0.32
V6	0.80	0.87	0.20	0.53	0.81	0.89	0.21	0.51

For PTB-XL (see Table 8 and Table 9), results are, overall, the worst, although some leads (namely V4, V5, and V6), due to higher correlation with the reference leads, are better reconstructed than with the PTB database. Visually, it is possible to observe that, despite occasional baseline offset and prevalent noise, both reference leads enable the approximate reconstruction of most of the set of twelve standard leads.

### Influence of medical conditions

As aforementioned, medical conditions may affect differently the various leads of an ECG signal. While this is the main motivation behind the quest to reconstruct missing leads it may also be one of the main hurdles. If the medical condition is somehow not evident in the input lead, the algorithm could be led to reconstruct the remaining leads incorrectly without the proper information on the respective medical condition.

**Table 10:** Average correlation results for PTB-XL conversion from lead II, using the U-Net with a shared encoder, according to medical condition class

Class	Converted leads										
	I	III	aVR	aVL	aVF	V1	V2	V3	V4	V5	V6
NORM	0.79	0.42	−0.39	−0.32	0.86	0.83	0.76	0.64	0.70	0.85	0.90
MI	0.65	0.47	−0.37	−0.38	0.76	0.75	0.65	0.56	0.54	0.68	0.77
STTC	0.71	0.41	−0.41	−0.37	0.80	0.79	0.69	0.58	0.59	0.78	0.84
CD	0.65	0.60	−0.31	−0.28	0.84	0.59	0.54	0.58	0.58	0.63	0.71
HYP	0.75	0.42	−0.40	−0.24	0.80	0.85	0.70	0.56	0.61	0.81	0.87

As such, we conducted a differential performance evaluation according to the existence and type of diagnosed medical conditions on the signals. To do this, we use the expert clinical annotations on the PTB-XL database and separate the results by the superclass labelling of each test sample. The average *r* results for each converted lead and each superclass are presented in Table 10 (using lead II as reference) and Table 11 (using lead I as reference).

**Table 11:** Average correlation results for PTB-XL conversion from lead I, using the U-Net with a shared encoder, according to medical condition class

Class	Converted leads										
	II	III	aVR	aVL	aVF	V1	V2	V3	V4	V5	V6
NORM	0.73	−0.23	−0.35	−0.66	0.23	0.79	0.73	0.68	0.69	0.84	0.85
MI	0.50	−0.15	−0.26	−0.57	0.036	0.67	0.58	0.47	0.38	0.56	0.61
STTC	0.57	−0.20	−0.36	−0.64	0.00	0.74	0.64	0.55	0.48	0.71	0.76
CD	0.40	−0.31	−0.11	−0.48	−0.06	0.35	0.38	0.42	0.39	0.52	0.54
HYP	0.65	−0.29	−0.37	−0.60	0.07	0.78	0.65	0.58	0.59	0.78	0.82

Overall, no dominant difference could be observed between the results with normal signals and the results with signals with medical conditions. Similarly, no specific medical condition superclass presents considerably different performance results.

### Diagnosis using reconstructed signals

To further evaluate the quality of the reconstructed lead signals, we conduct an experiment on medical diagnosis using original signals *vs*. reconstructed signals. The convolutional neural network proposed by Nguyen et al. [24] is adapted for the classification of PTB-XL’s five superclasses when given five-second ECG segments. The proposed architecture is faithfully followed in this work, with the exception of the use of five neurons on the last fully-connected layer.

The method is trained/validated on the first eighty per cent of Lead I segments from the PTB-XL dataset. Then, the superclass diagnosis performance is evaluated on the remaining twenty per cent Lead I signals. Afterwards, the corresponding Lead II signals are used to obtain reconstructed Lead I signals, following the aforementioned one-to-all U-Net architectures with either a shared encoder or individual encoders. These reconstructed Lead I signals are then classified by the trained diagnosis model.

The diagnosis model obtained accuracies of $54.13\%$ when using original signals, $45.71\%$ when using reconstructed signals with a shared encoder, and $42.03\%$ when using signals reconstructed with individual encoders. Balanced accuracy results were $46.58\%$, $37.56\%$, and $37.00\%$, respectively, which denotes some bias towards the majority class (NORM), despite the use of class weights during training.

## Discussion

### Architecture comparison

Regarding the explored architectures, the results seem to indicate that the skip-connections of the U-Net give it the capability to send more information (and at more resolution levels) from the encoder to the decoders, granting it more flexibility and ultimately better performance than the AE. The multi-resolution supervision of the LRN, expected to improve overall performance, appears to excessively draw the model’s attention away from the details, which resulted in worse performance.

### One-to-all leads conversion

While lead II ECG signals are generally better for medical diagnosis in clinical scenarios, lead I is becoming increasingly important. The widespread implementation of ECG acquisition equipment in smartwatches, fitness bands, and other gadgets for daily use allows for the collection of lead I signals. Combining these growing applications with robust conversion algorithms would enable the recovery of missing leads on wearables and empower the next generation of robust continuous health monitoring.

Considering the overall results presented earlier in this paper, no lead is perfect for converting all twelve standard leads. Hence, lead II should be chosen as reference input when aVF or V5-V6 are the most important leads for the application at hand. Lead I serves better as a reference when aVR, aVL, or V1-V2 are more important. Otherwise, other leads (such as lead III) should probably be explored. Nevertheless, the results show it is possible to nicely reconstruct several leads using only one input lead without temporal alignment.

At last, regarding the use of one shared encoder *vs*. individual encoders, results suggest that the additional flexibility of having multiple encoders is only beneficial up to a point, and the higher complexity ends up opening the door to overfitting and loss of robustness. As such, for this application, one should expect a shared encoder to be the best option, considering its higher simplicity, faster inference, and similar performance.

### Comparison with the state-of-the-art

When compared with the state-of-the-art baseline proposed by Grande-Fidalgo et al. [17], the proposed method attained considerably improved results in lead reconstruction from single-lead blindly-segmented signals. One can assume that, although the baseline’s simplistic model presents advantages in terms of lightweight operation and robustness to overfitting, single-lead information is not enough for it to achieve reliable interlead conversion.

The fact the baseline method reconstructs signals point-by-point, unable to analyse broader local context information, makes it hard to reconstruct the signal without already having data from more than one channel. On the other hand, using convolutional layers allows the proposed method to use broader local information as context to adequately learn to reconstruct signals using only one lead as reference.

### Cross-database evaluation

The cross-database evaluations consisted of the use of models trained on PTB data to reconstruct signals from different databases, namely INCART and PTB-XL. Throughout these experiments, considerably lower-quality reconstructions were obtained. This is as expected since PTB data was seen by the models during training and both the INCART and PTB-XL databases are arguably more challenging regarding signal noise and variability.

For either database, differences in acquisition settings and electrode placement result in inferior performance. The ideal solution is to always make sure the acquisition details of training and inference data match, to ensure optimal performance upon deployment. Nevertheless, the robustness in cross-database scenarios is a relevant issue that merits further research.

### Influence of medical conditions

Experiments were conducted on the reconstruction of signals with certain medical conditions. The results presented earlier show there was no considerable difference in reconstruction performance when using healthy signals *vs*. signals with medical conditions.

This is likely due to the presence of medical conditions on the PTB signals originally used for training the model. Thus, although the behaviour of the proposed methodology should be expected to vary slightly in the presence of medical conditions, it should not have a considerable impact on its baseline performance.

### Diagnosis using reconstructed signals

The last experiment consisted of using the deep learning model proposed by Nguyen et al. [24] for superclass diagnosis with original and reconstructed signals. The obtained results illustrate the limitations of the current methodology, as the reconstruction error propagates forward into the performance of diagnosis methods that may rely on the converted signals.

One should note that, according to the results presented earlier in this paper, Lead II to Lead I conversion is arguably not the most reliable, and reference lead choice should take into account the results presented in this work. Nevertheless, future efforts should be devoted to ensuring that, in spite of any reconstruction error, all useful signal information should be correctly reconstructed to not affect subsequent diagnosis performance.

## Conclusion

This work implemented and compared the performance of three deep learning architectures for interlead conversion of ECG signals. Unlike the literature, this work focused on the more challenging scenario of single-lead blindly-segmented inputs from limb leads. The proposed model was explored on 12-lead acquisitions from three different databases. Ablation studies were conducted on the architectures used for conversion and on the use of a shared encoder vs. individual encoders. Moreover, the model was evaluated on both single-database and cross-database scenarios, including an experiment on the effect of medical conditions on signal reconstruction and the study of diagnosis performance with original *vs*. converted signals.

Despite the considerably more challenging scenario, the proposed methodology based on a U-Net was capable of obtaining relatively good results. Each reference lead enabled the high-quality reconstruction of several of the twelve standard ECG leads, in some cases reaching state-of-the-art level performance. Both lead I and II appear to be especially suitable for certain sets of leads and could be used on specific target applications that focus on those.

In the cross-database scenario, despite the acquisition setup differences, results were promising especially with the INCART database. Finally, the analysis of the influence of medical conditions has shown no considerable effect of pathologies on the performance of the proposed methodology. However, a state-of-the-art methodology for automatic diagnosis revealed lower accuracy when using reconstructed signals, a problem that should be addressed in future research.

Although the results are promising, further efforts should be devoted to improving the methodologies for interlead conversion using single-lead blindly-segmented inputs. Namely, the pre-processing and normalisation of the signals, as well as the robustness to diverse acquisition setups, should be the target of further research. Additionally, task-oriented objective functions should be explored to ensure useful signal information is kept and avoid performance losses in subsequent diagnoses.

With some consolidation, the proposed methodology could be the key to better cardiac health monitoring in wearable devices and less obtrusive clinical scenarios. Taking the example of emergency rooms, if we can retrieve all twelve leads (or the most important among these) from Lead I signals, then patients will only need two electrodes placed on the wrists to have their ECG collected, instead of the full set of 10 electrodes on wrists, ankles, and chest. This is a meaningful step towards higher comfort and usability for both patients in clinical settings and users in other scenarios involving the monitoring of ECG signals. Additionally, albeit outside the scope of this work, the proposed methodology for interlead conversion could also be applicable to other multi-channel signals where the different channels correspond to different perspectives over the same physiological phenomenon.

## Supplementary information

**Additional file 1: Fig. S1: **Results of cross-database INCART reconstruction from lead II. Example cross-database result of lead II to all conversion on the INCART dataset (each row depicts one converted. lead, with the shared encoder on the left column and individual encoders in the right column; the horizontal axis represents time, while the vertical axis corresponds to the normalised signal amplitude).

**Additional file 2: Fig. S2:**Results of cross-database INCART reconstruction from lead I. Example cross-database result of lead I to all conversion on the INCART dataset (each row depicts one converted lead, with the shared encoder on the left column and individual encoders in the right column; the horizontal axis represents time, while the vertical axis corresponds to the normalised signal amplitude).

**Additional file 3: Fig. S3:**Results of cross-database PTB-XL reconstruction from lead II. Example cross-database result of lead II to all conversion on the PTB-XL dataset(each row depicts one converted lead, with the shared encoder on the left column and individual encoders in the right column; the horizontal axis represents time, while the vertical axis corresponds to the normalised signal amplitude).

**Additional file 4: Fig. S4:**Results of cross-database PTB-XL reconstruction from lead I. (each row depicts one converted lead, with the shared encoder on the left column and individual encoders in the right column; the horizontal axis represents time , while the vertical axis corresponds to the normalised signal amplitude).

**Additional file 5: Fig. S5 ** Training evolution from lead II, with individual encoders. Training loss evolution for the individual encoders model with lead II as reference.

**Additional file 6: Fig. S6:**Training evolution from lead II, with shared encoder. Training loss evolution for the shared encoders model with lead II as reference.

**Additional file 7: Fig. S7:**Training evolution from lead I, with individual encoders. Training loss evolution for the individual encoders model with lead I as reference.

**Additional file 8: Fig. S8:**Training evolution from lead I, with shared encoder. Training loss evolution for the shared encoders model with lead I as reference.

## Data Availability

The PTB, INCART, and PTB-XL databases used in this work are publicly available at Physionet: https://www.physionet.org/about/database/. Code is available at https://github.com/jtrpinto/ecg-conversion.

## Abbreviations

AE: autoencoder
CD: conduction disturbance
ECG: electrocardiogram
FTDNN: focused time-delay neural network
GAN: generative adversarial network
HYP: hypertrophy
LRN: label refinement network
LSTM: long short-term memory
MI: myocardial infarction
NORM: normal electrocardiogram
RMSE: root mean square error
SSIM: Structural Similarity Index Measure
STTC: ST/T change.

## Footnotes

1 ECG conversion code available at: https://github.com/jtrpinto/ecg-conversion.

2 Examples were selected among all test samples to correspond to the median overall *r* result for each scenario. Hence, they represent an overall median result, and the methodology should be expected to offer better results in half of the occasions.

3 Processing times were computed on an NVidia GTX 1080 GPU.
