# Supplementary material for: Electrocardiogram lead conversion from single-lead blindly-segmented signals
Source: BMC Med Inform Decis Mak. 2022 Nov 29;22:314. doi: 10.1186/s12911-022-02063-6 (PMC9710059; doi:10.1186/s12911-022-02063-6)
Supplement: Supplementary file 1 — Additional file 1: Fig. S1: Results of cross-database INCART reconstruction from lead II. Example cross-database result of lead II to all conversion on the INCART dataset (each row depicts one converted. lead, with the shared encoder on the left column and individual encoders in the right column; the horizontal axis represents time, while the vertical axis corresponds to the normalised signal amplitude). [file 12911_2022_2063_MOESM1_ESM.pdf]

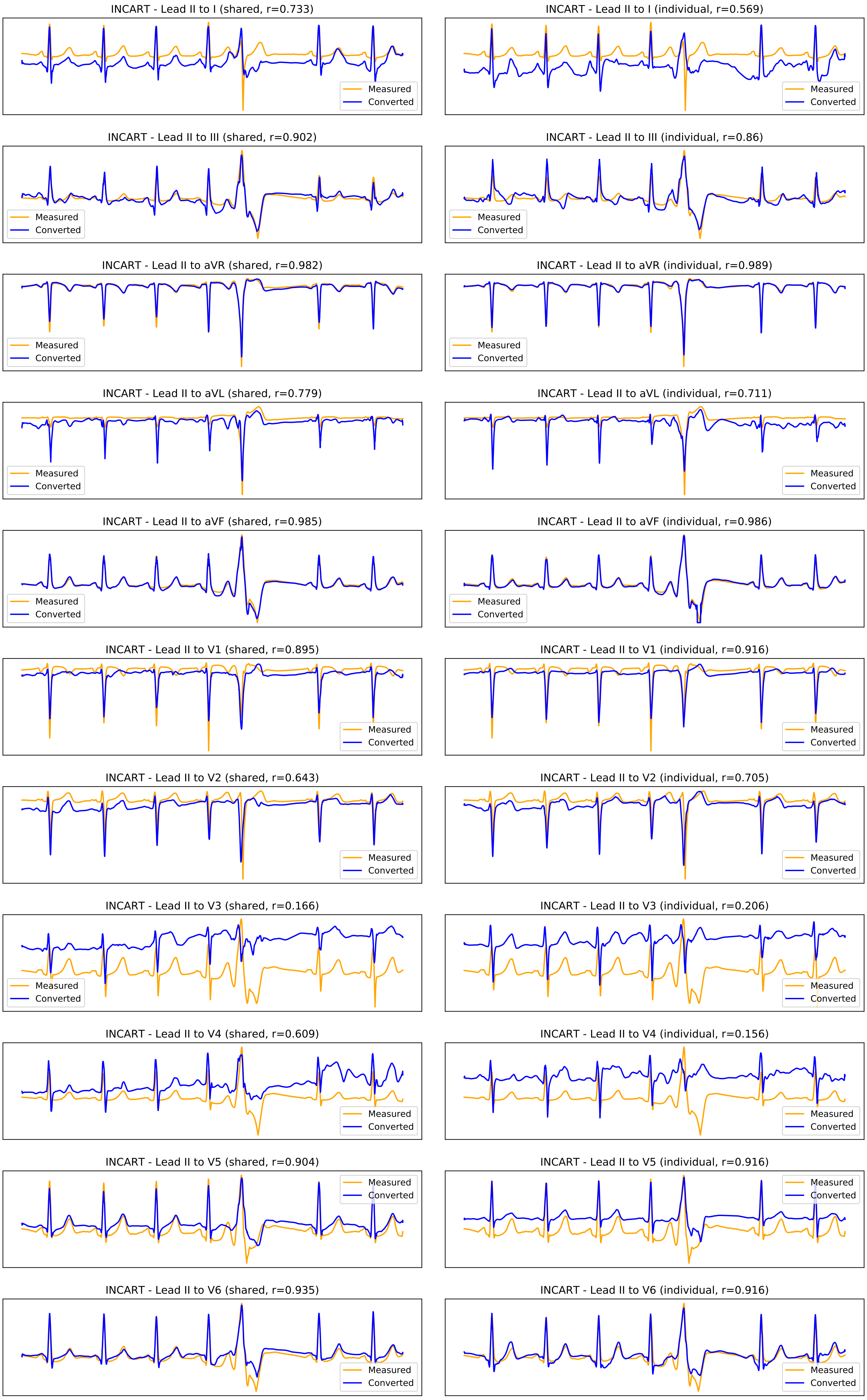

INCART - Lead II to I (shared, r=0.733)
Measured
Converted
INCART - Lead II to I (individual, r=0.569)
Measured
Converted
INCART - Lead II to III (shared, r=0.902)
Measured
Converted
INCART - Lead II to III (individual, r=0.86)
Measured
Converted
INCART - Lead II to aVR (shared, r=0.982)
Measured
Converted
INCART - Lead II to aVR (individual, r=0.989)
Measured
Converted
INCART - Lead II to aVL (shared, r=0.779)
Measured
Converted
INCART - Lead II to aVL (individual, r=0.711)
Measured
Converted
INCART - Lead II to aVF (shared, r=0.985)
Measured
Converted
INCART - Lead II to aVF (individual, r=0.986)
Measured
Converted
INCART - Lead II to V1 (shared, r=0.895)
Measured
Converted
INCART - Lead II to V1 (individual, r=0.916)
Measured
Converted
INCART - Lead II to V2 (shared, r=0.643)
Measured
Converted
INCART - Lead II to V2 (individual, r=0.705)
Measured
Converted
INCART - Lead II to V3 (shared, r=0.166)
Measured
Converted
INCART - Lead II to V3 (individual, r=0.206)
Measured
Converted
INCART - Lead II to V4 (shared, r=0.609)
Measured
Converted
INCART - Lead II to V4 (individual, r=0.156)
Measured
Converted
INCART - Lead II to V5 (shared, r=0.904)
Measured
Converted
INCART - Lead II to V5 (individual, r=0.916)
Measured
Converted
INCART - Lead II to V6 (shared, r=0.935)
Measured
Converted
INCART - Lead II to V6 (individual, r=0.916)
Measured
Converted
