# Supplementary material for: Electrocardiogram lead conversion from single-lead blindly-segmented signals
Source: BMC Med Inform Decis Mak. 2022 Nov 29;22:314. doi: 10.1186/s12911-022-02063-6 (PMC9710059; doi:10.1186/s12911-022-02063-6)
Supplement: Supplementary file 2 — Additional file 2: Fig. S2:Results of cross-database INCART reconstruction from lead I. Example cross-database result of lead I to all conversion on the INCART dataset (each row depicts one converted lead, with the shared encoder on the left column and individual encoders in the right column; the horizontal axis represents time, while the vertical axis corresponds to the normalised signal amplitude). [file 12911_2022_2063_MOESM2_ESM.pdf]

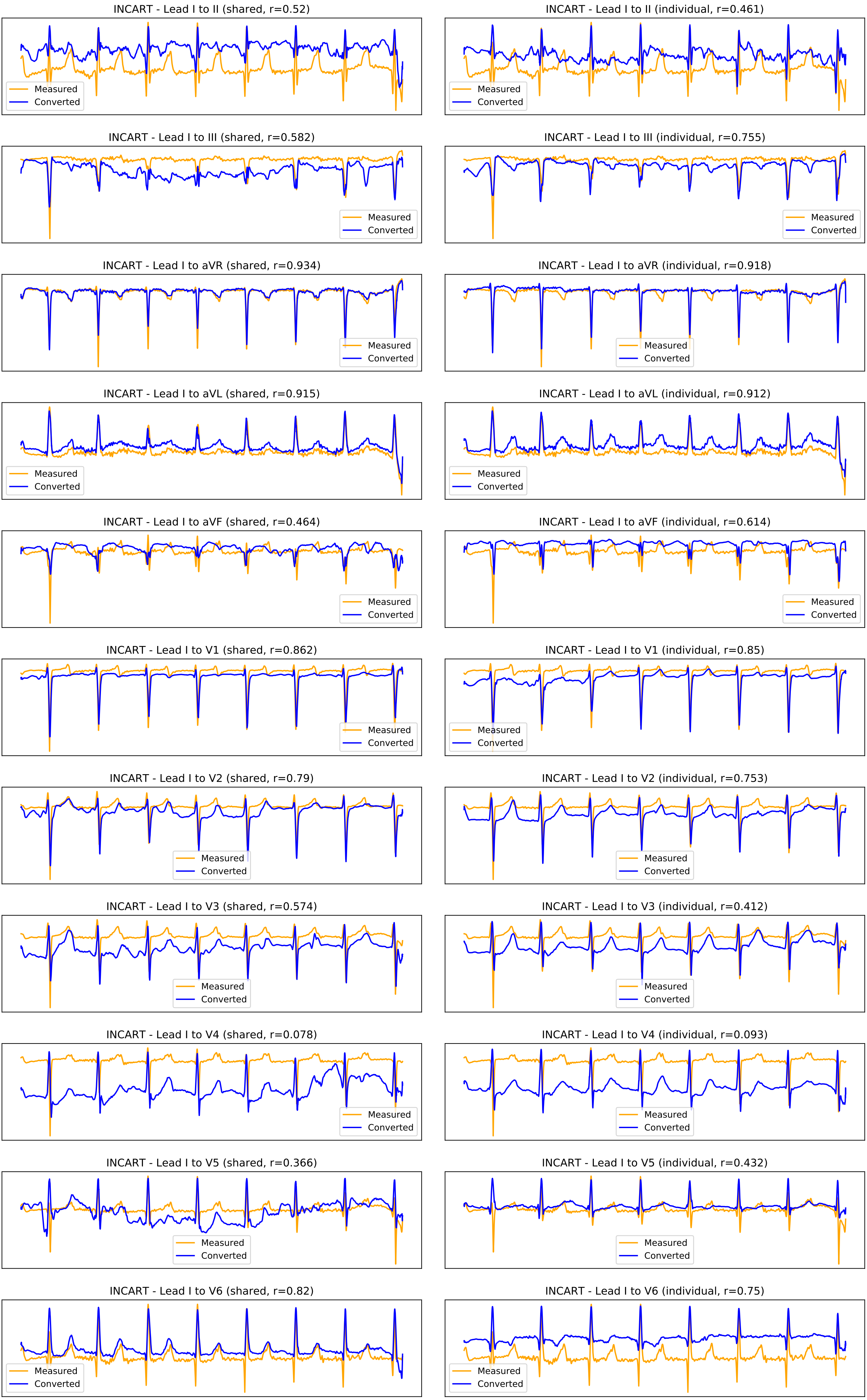
INCART - Lead I to II (shared, r=0.52)
Measured
Converted
INCART - Lead I to II (individual, r=0.461)
Measured
Converted
INCART - Lead I to III (shared, r=0.582)
Measured
Converted
INCART - Lead I to III (individual, r=0.755)
Measured
Converted
INCART - Lead I to aVR (shared, r=0.934)
Measured
Converted
INCART - Lead I to aVR (individual, r=0.918)
Measured
Converted
INCART - Lead I to aVL (shared, r=0.915)
Measured
Converted
INCART - Lead I to aVL (individual, r=0.912)
Measured
Converted
INCART - Lead I to aVF (shared, r=0.464)
Measured
Converted
INCART - Lead I to aVF (individual, r=0.614)
Measured
Converted
INCART - Lead I to V1 (shared, r=0.862)
Measured
Converted
INCART - Lead I to V1 (individual, r=0.85)
Measured
Converted
INCART - Lead I to V2 (shared, r=0.79)
Measured
Converted
INCART - Lead I to V2 (individual, r=0.753)
Measured
Converted
INCART - Lead I to V3 (shared, r=0.574)
Measured
Converted
INCART - Lead I to V3 (individual, r=0.412)
Measured
Converted
INCART - Lead I to V4 (shared, r=0.078)
Measured
Converted
INCART - Lead I to V4 (individual, r=0.093)
Measured
Converted
INCART - Lead I to V5 (shared, r=0.366)
Measured
Converted
INCART - Lead I to V5 (individual, r=0.432)
Measured
Converted
INCART - Lead I to V6 (shared, r=0.82)
Measured
Converted
INCART - Lead I to V6 (individual, r=0.75)
Measured
Converted
