# Supplementary material for: Electrocardiogram lead conversion from single-lead blindly-segmented signals
Source: BMC Med Inform Decis Mak. 2022 Nov 29;22:314. doi: 10.1186/s12911-022-02063-6 (PMC9710059; doi:10.1186/s12911-022-02063-6)
Supplement: Supplementary file 3 — Additional file 3: Fig. S3:Results of cross-database PTB-XL reconstruction from lead II. Example cross-database result of lead II to all conversion on the PTB-XL dataset(each row depicts one converted lead, with the shared encoder on the left column and individual encoders in the right column; the horizontal axis represents time, while the vertical axis corresponds to the normalised signal amplitude). [file 12911_2022_2063_MOESM3_ESM.pdf]

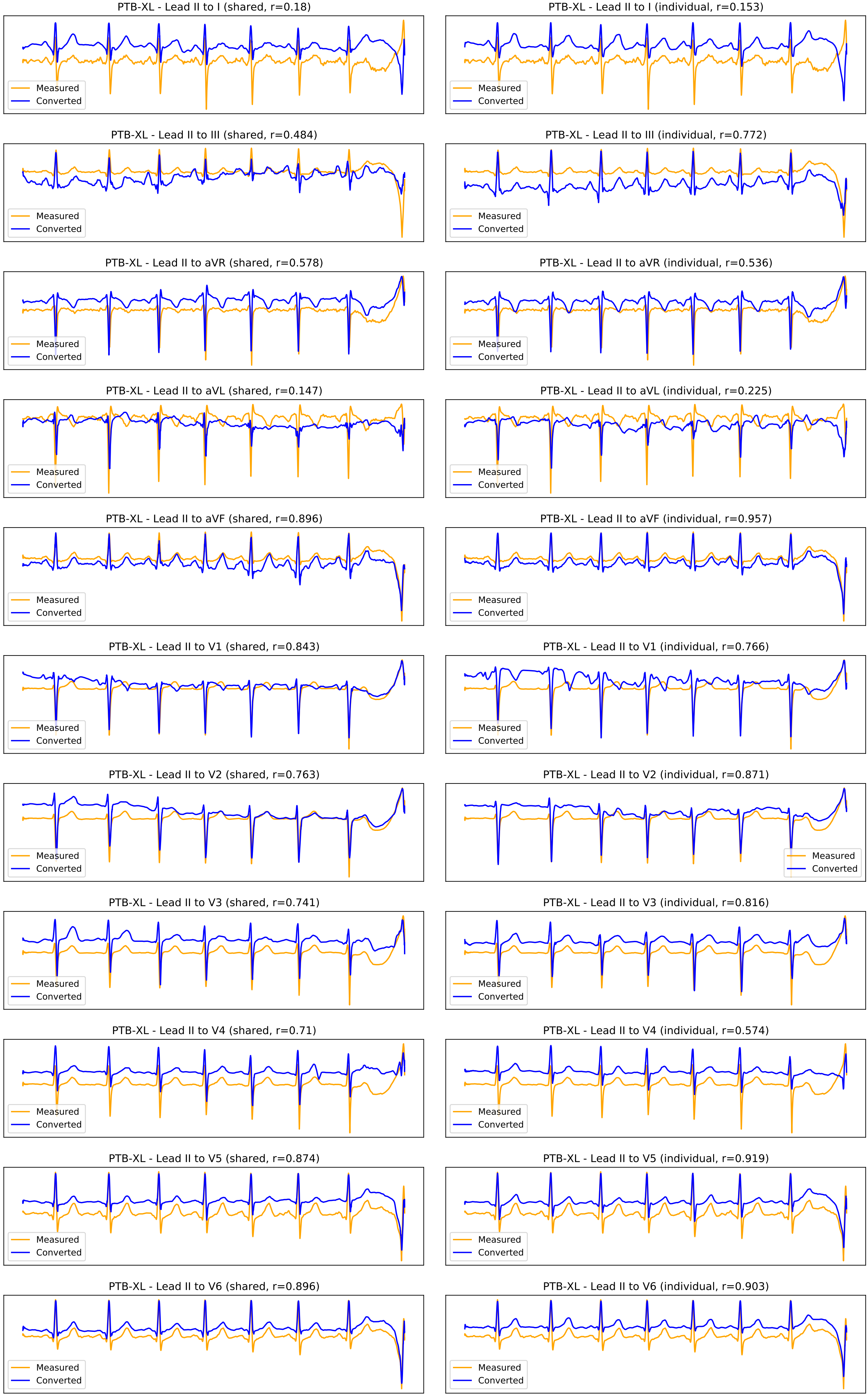
PTB-XL - Lead II to I (shared, r=0.18)
PTB-XL - Lead II to I (individual, r=0.153)
PTB-XL - Lead II to III (shared, r=0.484)
PTB-XL - Lead II to III (individual, r=0.772)
PTB-XL - Lead II to aVR (shared, r=0.578)
PTB-XL - Lead II to aVR (individual, r=0.536)
PTB-XL - Lead II to aVL (shared, r=0.147)
PTB-XL - Lead II to aVL (individual, r=0.225)
PTB-XL - Lead II to aVF (shared, r=0.896)
PTB-XL - Lead II to aVF (individual, r=0.957)
PTB-XL - Lead II to V1 (shared, r=0.843)
PTB-XL - Lead II to V1 (individual, r=0.766)
PTB-XL - Lead II to V2 (shared, r=0.763)
PTB-XL - Lead II to V2 (individual, r=0.871)
PTB-XL - Lead II to V3 (shared, r=0.741)
PTB-XL - Lead II to V3 (individual, r=0.816)
PTB-XL - Lead II to V4 (shared, r=0.71)
PTB-XL - Lead II to V4 (individual, r=0.574)
PTB-XL - Lead II to V5 (shared, r=0.874)
PTB-XL - Lead II to V5 (individual, r=0.919)
PTB-XL - Lead II to V6 (shared, r=0.896)
PTB-XL - Lead II to V6 (individual, r=0.903)
Measured
Converted
