# Supplementary material for: Electrocardiogram lead conversion from single-lead blindly-segmented signals
Source: BMC Med Inform Decis Mak. 2022 Nov 29;22:314. doi: 10.1186/s12911-022-02063-6 (PMC9710059; doi:10.1186/s12911-022-02063-6)
Supplement: Supplementary file 4 — Additional file 4: Fig. S4:Results of cross-database PTB-XL reconstruction from lead I. (each row depicts one converted lead, with the shared encoder on the left column and individual encoders in the right column; the horizontal axis represents time , while the vertical axis corresponds to the normalised signal amplitude). [file 12911_2022_2063_MOESM4_ESM.pdf]

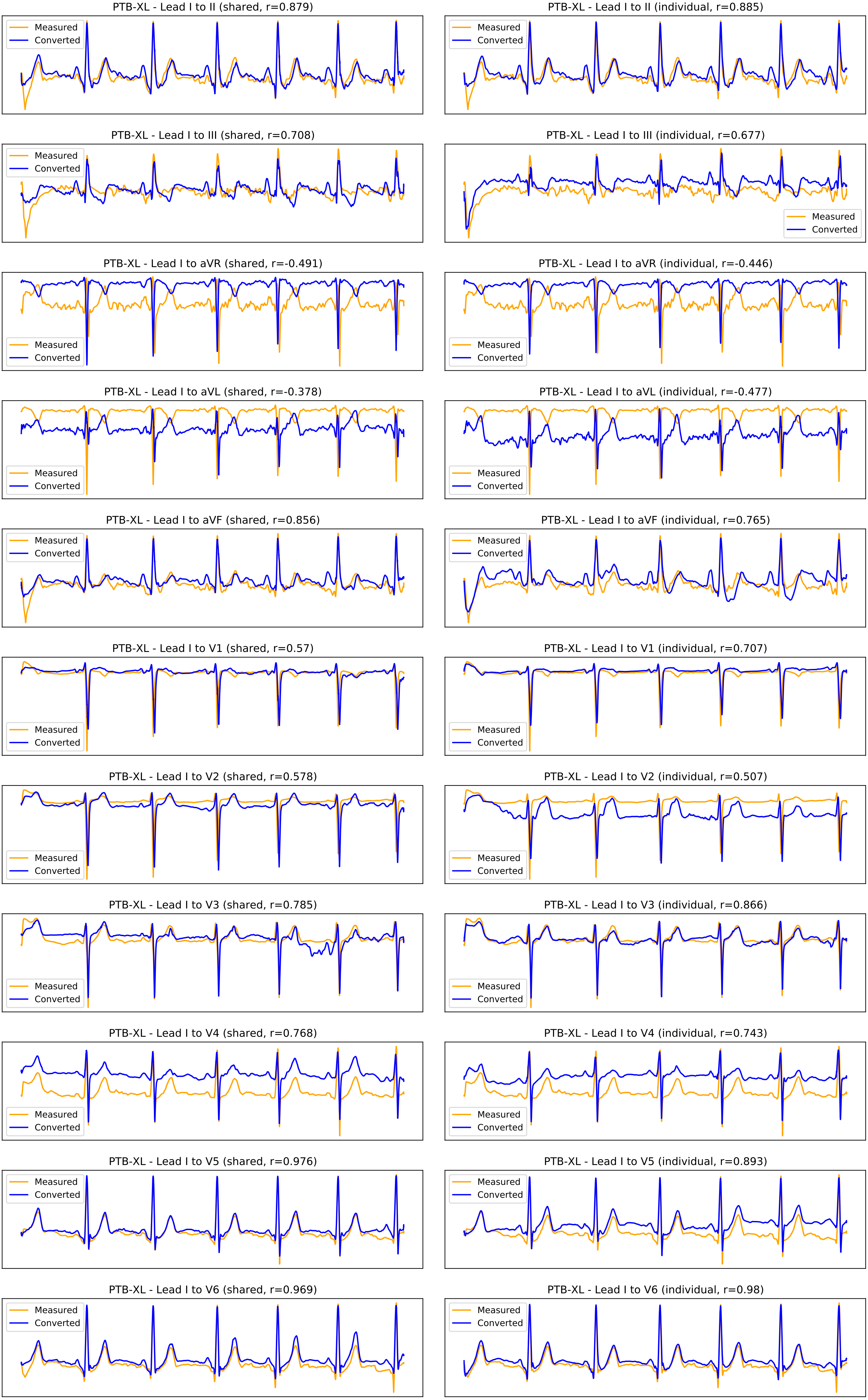
PTB-XL - Lead I to II (shared, r=0.879)
PTB-XL - Lead I to II (individual, r=0.885)
PTB-XL - Lead I to III (shared, r=0.708)
PTB-XL - Lead I to III (individual, r=0.677)
PTB-XL - Lead I to aVR (shared, r=-0.491)
PTB-XL - Lead I to aVR (individual, r=-0.446)
PTB-XL - Lead I to aVL (shared, r=-0.378)
PTB-XL - Lead I to aVL (individual, r=-0.477)
PTB-XL - Lead I to aVF (shared, r=0.856)
PTB-XL - Lead I to aVF (individual, r=0.765)
PTB-XL - Lead I to V1 (shared, r=0.57)
PTB-XL - Lead I to V1 (individual, r=0.707)
PTB-XL - Lead I to V2 (shared, r=0.578)
PTB-XL - Lead I to V2 (individual, r=0.507)
PTB-XL - Lead I to V3 (shared, r=0.785)
PTB-XL - Lead I to V3 (individual, r=0.866)
PTB-XL - Lead I to V4 (shared, r=0.768)
PTB-XL - Lead I to V4 (individual, r=0.743)
PTB-XL - Lead I to V5 (shared, r=0.976)
PTB-XL - Lead I to V5 (individual, r=0.893)
PTB-XL - Lead I to V6 (shared, r=0.969)
PTB-XL - Lead I to V6 (individual, r=0.98)
Measured
Converted
