# Supplementary figures and images for: Electrocardiogram lead conversion from single-lead blindly-segmented signals
Source: BMC Med Inform Decis Mak. 2022 Nov 29;22:314. doi: 10.1186/s12911-022-02063-6 (PMC9710059; doi:10.1186/s12911-022-02063-6)

Training Evolution (Lead II to all, individual encoder)

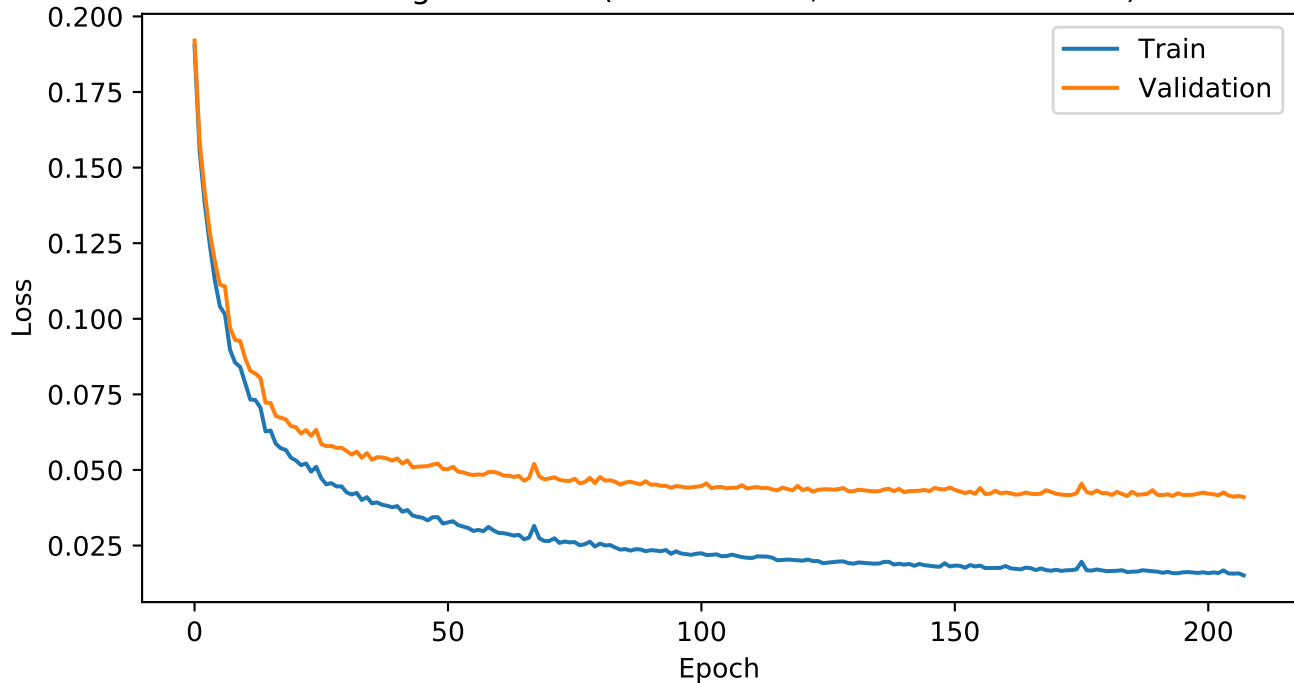

Supplement: Supplementary file 5 — Additional file 5: Fig. S5 Training evolution from lead II, with individual encoders. Training loss evolution for the individual encoders model with lead II as reference. [file 12911_2022_2063_MOESM5_ESM.pdf]

Training Evolution (Lead II to all, shared encoder)

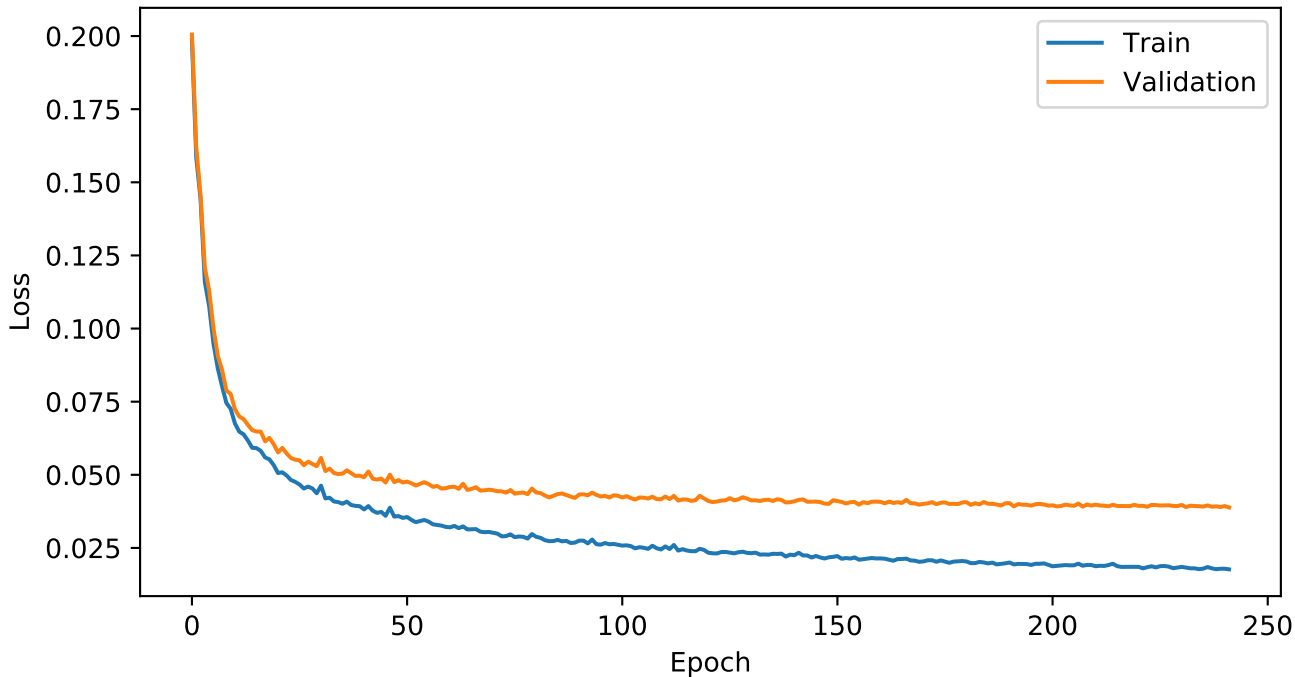

Supplement: Supplementary file 6 — Additional file 6: Fig. S6:Training evolution from lead II, with shared encoder. Training loss evolution for the shared encoders model with lead II as reference. [file 12911_2022_2063_MOESM6_ESM.pdf]

Training Evolution (Lead I to all, individual encoder)

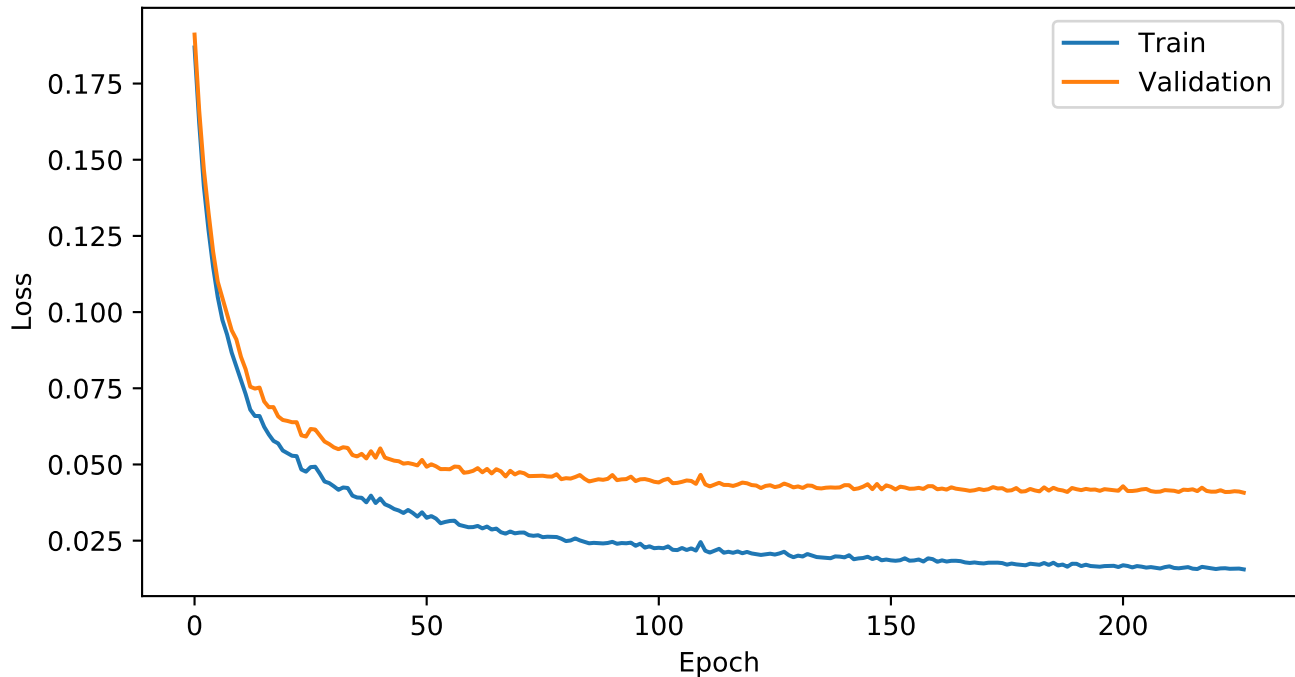

Supplement: Supplementary file 7 — Additional file 7: Fig. S7:Training evolution from lead I, with individual encoders. Training loss evolution for the individual encoders model with lead I as reference. [file 12911_2022_2063_MOESM7_ESM.pdf]

Training Evolution (Lead I to all, shared encoder)

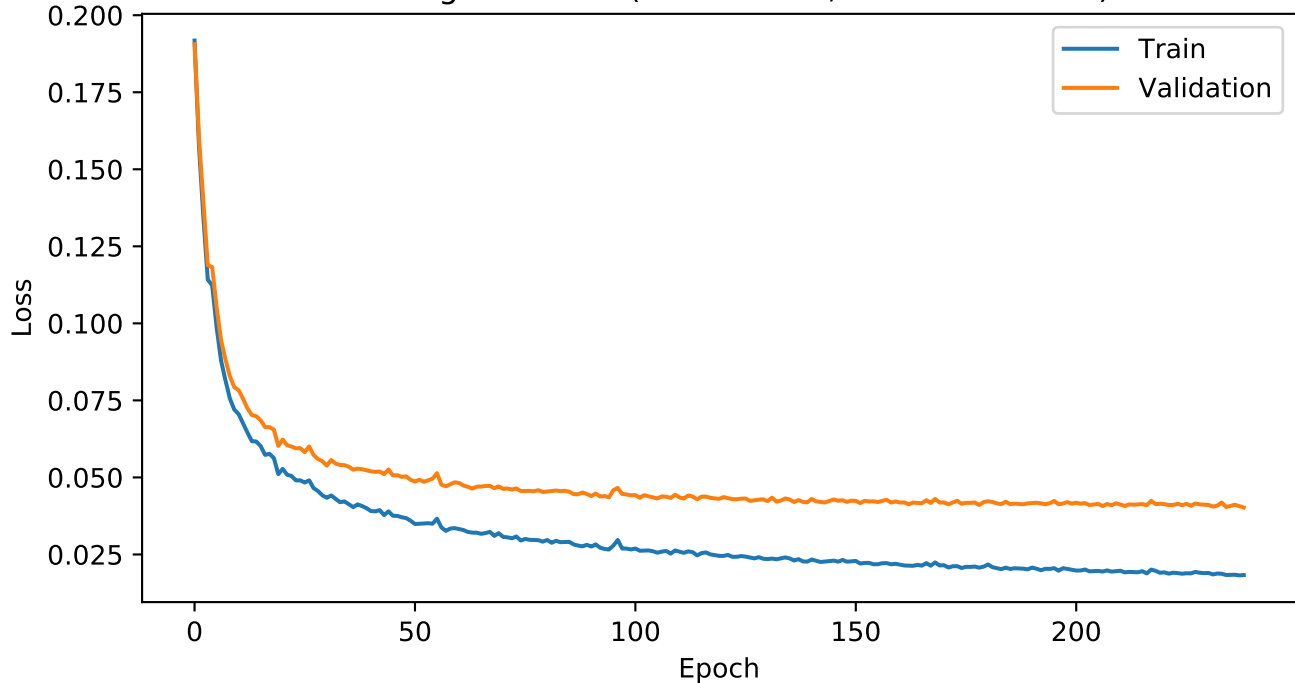

Supplement: Supplementary file 8 — Additional file 8: Fig. S8:Training evolution from lead I, with shared encoder. Training loss evolution for the shared encoders model with lead I as reference. [file 12911_2022_2063_MOESM8_ESM.pdf]
